# Influenza A(H1N1)pdm09 Virus Infection in Giant Pandas, China

**DOI:** 10.3201/eid2003.131531

**Published:** 2014-03

**Authors:** Desheng Li, Ling Zhu, Hengmin Cui, Shanshan Ling, Shengtao Fan, Zhijun Yu, Yuancheng Zhou, Tiecheng Wang, Jun Qian, Xianzhu Xia, Zhiwen Xu, Yuwei Gao, Chengdong Wang

**Affiliations:** Key Laboratory of Animal Disease and Human Health, College of Veterinary Medicine of Sichuan Agricultural University, Ya’an, People’s Republic of China (D. Li, L. Zhu, H. Cui, Z. Xu);; Key Laboratory of Animal Biotechnology Center of Sichuan Province, College of Veterinary Medicine of Sichuan Agricultural University, Ya’an (D. Li, L. Zhu, Y. Zhou, Z. Xu, C. Wang);; China Conservation and Research Center for the Giant Panda, Ya’an (D. Li, S. Ling, C. Wang);; Research Center of Wildlife Disease, Key Laboratory of Jilin Province for Zoonosis Prevention and Control, Military Veterinary Research Institute of Academy of Military Medical Sciences, Changchun, People’s Republic of China (T. Wang, J. Qian, X. Xia, Y. Gao);; Institute of Laboratory Animal Sciences, Chinese Academy of Medical Sciences, and Peking Union Medical College, Beijing, People’s Republic of China (S. Fan, Z. Yu)

**Keywords:** giant pandas, pandemic, H1N1, influenza A virus, influenza A(H1N1)pdm09, serologic analysis, influenza, viruses, China, Ailuropoda melanoleuca, respiratory infections

## Abstract

We confirmed infection with influenza A(H1N1)pdm09 in giant pandas in China during 2009 by using virus isolation and serologic analysis methods. This finding extends the host range of influenza viruses and indicates a need for increased surveillance for and control of influenza viruses among giant pandas.

In April 2009, the Centers for Disease Control and Prevention reported the emergence of a novel strain of influenza A(H1N1) virus, which is now referred to as influenza A(H1N1)pdm09 or pH1N1. This virus rapidly affected countries worldwide and continues to circulate as a seasonal influenza virus. In addition to widespread infection of humans, reported have been published of pH1N1 virus infection in domestic and nondomestic animals, including cats, dogs, ferrets, swine, and several wildlife species (*1*–*4*). Here, we report a confirmed case of pH1N1 virus infection in giant pandas (*Ailuropoda melanoleuca*) in China.

## The Study

During the human outbreak of pH1N1 in China, 3 giant pandas at the Conservation and Research Center for the Giant Panda in Ya’an City, Sichuan Province, showed clinical signs suggestive of a respiratory tract infection, including pyrexia, anorexia, malaise, conjunctivitis, and sneezing. Nasal swab specimens were successfully collected under anesthesia from 1 of the affected pandas (Ximeng). All 3 pandas recovered after receiving 75 mg of oseltamivir phosphate twice daily for 5–6 days. For serologic analysis, serum samples were collected from all 3 pandas ≈3 months after the resolution of the respiratory illness; a serum sample collected before onset of the respiratory illness was also available for all 3 animals.

The nasal swab specimens were collected in 1 mL phosphate-buffered saline and tested for evidence of pH1N1 virus and several other pathogens reported (*5*–*7*) or suspected to occur in giant pandas: canine distemper virus, canine adenovirus, canine coronavirus, canine herpesvirus, and canine parainfluenza virus. Testing for detection of influenza A virus was performed by using a real-time reverse transcription PCR method, as described by the World Health Organization (WHO) (*8*); other pathogens were tested by different PCR methods.

RNA from the swab specimens tested positive for the hemagglutinin gene of pH1N1 virus. No other pathogens were detected.

**Figure 1:**
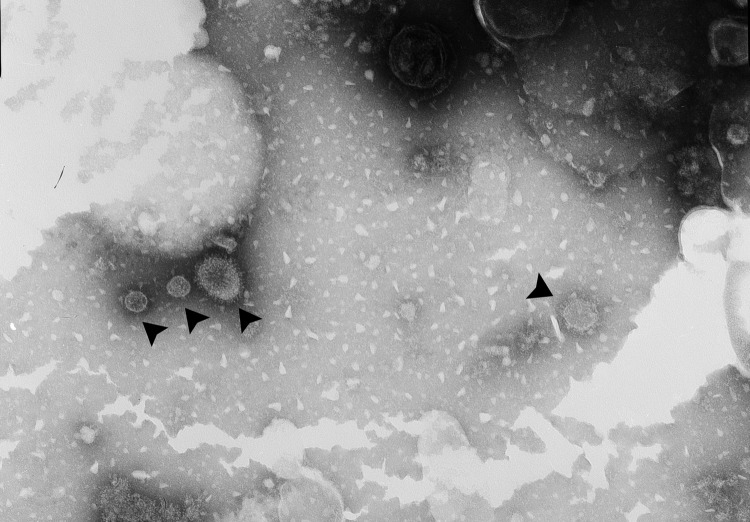
Negative-staining electron micrograph image showing influenza A(H1N1)pdm09 virus particles (arrowheads) in allantoic fluid supernatant collected from specific pathogen free eggs after injection with a nasal swab sample collected from a giant panda in China. Original magnification ×40,000.

To isolate and characterize the pH1N1 virus, we injected 10-day-old specific pathogen free embryonated chicken eggs with material collected from one of the nasal swab samples. Allantoic fluid from the injected eggs agglutinated 0.5% (vol/vol) chicken erythrocytes, indicating the presence of replication-competent virus in the nasal swab sample. When evaluated by electron microscopy, allantoic fluid supernatant from the infected egg displayed enveloped influenza virus–like particles of 100–120 nm (Figure 1).

**Figure 2:**
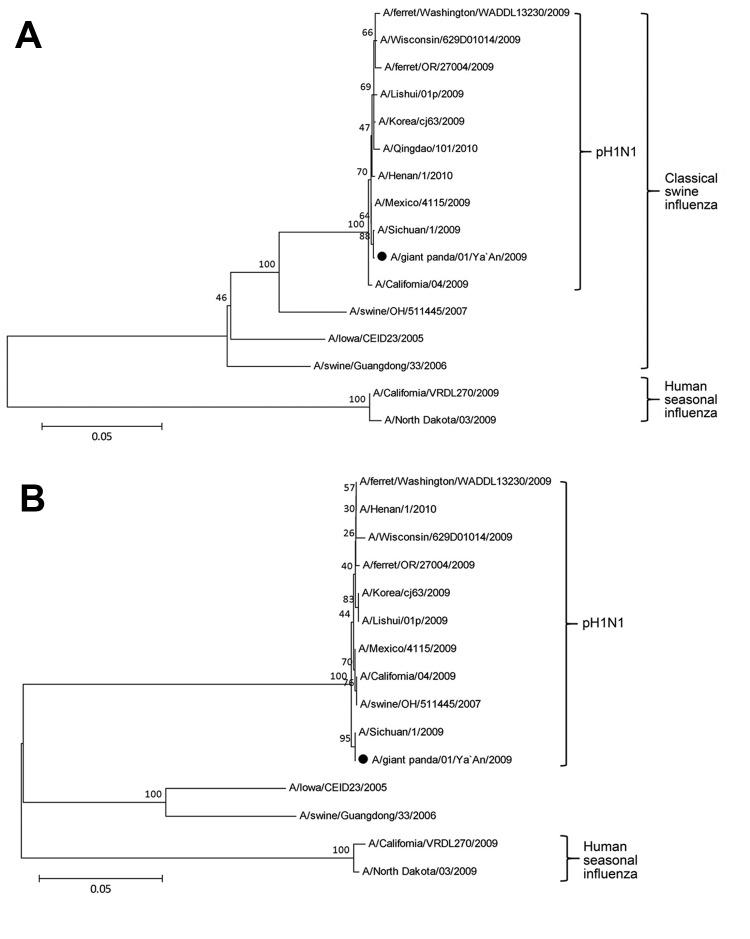
Phylogenetic trees of influenza A(H1N1)pdm09 virus (pH1N1) isolated from a giant panda in China compared with previously characterized pandemic influenza A(H1N1) viruses. A) Hemagglutinin gene nucleotide sequences; B) neuraminidase gene nucleotide sequences. Neighbor-joining trees were created by using MegAlign software version 5.0 (www.megasoftware.net). Bootstrapping with 1,000 replicates was performed to determine the percentage reliability for each internal node. Horizontal branch lengths are proportional to genetic distances. Black dot indicates the isolate from this study. Scale bars indicate nucleotide substitutions per site.

We sequenced the entire genome of the virus by using RNA harvested from the allantoic fluid of infected eggs. Sequence analysis was performed as described by WHO by using methods for pH1N1 virus (*9*). A BLAST search (http://blast.ncbi.nlm.nih.gov/Blast.cgi) was used to identify sequences similar to those of the giant panda isolate; these sequences were GenBank accession nos. KF277197–KF277204. Analyses showed that each of the 8 gene segments of the virus we isolated were closely related to pH1N1 viruses circulating among humans, including a human representative strain (A/California/04/2009) and a contemporary strain (A/Sichuan/1/2009); these viruses showed 98.6%–100% nt identity to the panda strain (Technical Appendix Table 1). Phylogenetic analysis of the 8 gene segments of the virus we isolated showed that the isolate was related to the pH1N1 virus clade of influenza viruses (Figure 2; Technical Appendix Figure). The results of the BLAST and phylogenetic analyses suggest that a human pH1N1 virus was transmitted directly to giant pandas without recombination or significant adaptation. We named the virus that we isolated A/giant panda/01/Ya’an/2009 (H1N1).

A/giant panda/01/Ya’an/2009 (H1N1) virus contained glutamine at hemagglutinin amino acid position 226 (H3 numbering), which has been found in human pH1N1 isolates and confers binding to human cell-surface receptors (*10*). In addition, alanine at position 271 of polymerase protein 2 of the isolate reported here has been shown to be required for respiratory droplet–mediated transmission of A/Sichuan/01/2009 virus (*10*). Thus, the genomic sequences of the giant panda isolate appear to have many features known to be important for the replication and transmission of pH1N1 viruses in other mammalian species.

Serum samples collected from the affected pandas before their illnesses and 3 months after the resolution of the illnesses were tested for the presence of hemagglutination inhibition (HI) antibodies against a panel of influenza A viruses. HI antibody titers were measured against the A/giant panda/01/Ya’an/2009 (H1N1) isolate, human seasonal influenza (H3N2) virus (A/Victoria/361/2011), avian (H5N1) virus (A/duck/Anhui/1/2006), avian (H6N1) virus (A/Mallard/SanJiang/275/2007), and avian (H7N1) virus (A/Baer’s pochard/HuNan/414/2010). All 3 pandas had high titers of HI antibodies against the pH1N1 virus 3 months after illness (Table).

**Table:** Hemagglutination inhibition antibody titers against influenza A viruses in serum samples collected from giant pandas before and after respiratory infection, China, 2009*

Time	Animal name	Date collected	Titer					
			A(H1N1)pdm09	H3	H6	H5	H7	H9
Before infection	Ximeng	2004 Oct 5	<10	ND	ND	<10	ND	ND
	Gege	2009 Jul 29	<10	<10	<10	<10	<10	<10
	Zhangka	2009 Dec 1	320	80	<10	<10	<10	<10
After infection	Ximeng	2010 Mar 23	640	<10	<10	<10	<10	<10
	Gege	2010 Mar 31	640	<10	80	<10	<10	<10
	Zhangka	2010 Mar 27	640	80	40	<10	<10	<10

*All serum samples were pretreated with *Vibrio cholerae* receptor–destroying enzyme (Sigma-Aldrich, St. Louis, MO, USA) before testing. ND, not done.

One panda (Zhangka) had detectable pH1N1 and H3 subtype HI antibodies before infection; this serum sample was collected before the display of overt clinical signs in the animal but after the onset of the human pH1N1 outbreak in China. It is unknown if these antibodies reflect previous exposure to pH1N1 virus or are reflective of cross-reactive antibodies generated against antigenic sites in previously circulating influenza viruses.

Two of the pandas (Gege and Zhangka) also had serum antibodies that inhibited hemagglutination mediated by an H6 subtype avian influenza virus 3 months after the respiratory infection, whereas no such antibodies were detected before the respiratory infection (Table). When reference serum samples known to contain HI antibodies against each of the viral subtypes were evaluated for potential cross-reactivity against the other influenza subtypes, we observed no apparent cross-reactivity of pH1N1 antibodies against the H6 subtype virus (Technical Appendix Table 2). Therefore, although it is possible that the high titers of HI antibodies against pH1N1 virus in these animals cross-reacted with the H6 subtype viruses, we cannot exclude the possibility that these antibodies were generated in response to an independent exposure to an H6 influenza virus.

## Conclusions

Influenza A(H1N1)pdm09 virus infection has been found in mammals and birds of a variety of species since the virus was identified in early 2009 (*1*–*4*). We documented infection with this virus in giant pandas in China during 2009. Sequence analysis of a virus isolated from a nasal swab sample revealed that it was nearly identical to the pH1N1 virus circulating in humans, which suggests that the virus may have been transmitted to the pandas directly from humans. Furthermore, serologic analysis of samples from the affected pandas was consistent with productive infection by pH1N1 virus. Seroconversion was documented in each animal, although 1 animal appeared to have been previously exposed to pH1N1 or a related virus that was able to elicit cross-reactive antibodies. Similarly, some pandas had detectable HI antibodies against H3 and H6 subtype influenza viruses, raising the possibility that giant pandas may be infected with human- and avian-origin influenza viruses.

Only 1,600 wild and 300 captive giant pandas remain worldwide (*11*). Our findings highlight the risk posed by influenza viruses to these animals, extend the known host range of influenza A viruses, and have implications for wildlife conservation and influenza virus epidemiology. When taken together with previous reports of pH1N1 infection of other mammals, our data suggest that this virus can efficiently infect a wider range of mammalian species than can other influenza viruses. Increased surveillance and control of influenza viruses in giant panda populations are imperative for successful conservation of these endangered animals.

Technical Appendix Nucleotide identity of influenza A(H1N1)pdm09 virus isolated from a giant panda compared with other influenza viruses; antibody titers of reference serum sample against influenza viruses of different subtypes; and phylogenetic trees of PB2, PB 1, PA, NP, M, and NS genes of the isolate from this study compared with previously characterized viruses.
