# Supplementary material for: Influenza A(H1N1)pdm09 Virus Infection in Giant Pandas, China
Source: Emerg Infect Dis. 2014 Mar;20(3):480–3. doi: 10.3201/eid2003.131531 (PMC3944863; doi:10.3201/eid2003.131531)
Supplement: Technical Appendix — Nucleotide identity of influenza A(H1N1)pdm09 virus isolated from a giant panda compared with other influenza viruses; antibody titers of reference serum sample against influenza viruses of different subtypes; and phylogenetic trees of PB2, PB 1, PA, NP, M, and NS genes of the isolate from this study compared with previously characterized viruses. [file 13-1531-Techapp-s1.pdf]

Article DOI: http://dx.doi.org/10.3201/eid2003.131531

Influenza A(H1N1)pdm09 Virus Infection in Giant Pandas, China

Technical Appendix

Technical Appendix Table 1. Nucleotide identity of influenza A(H1N1)pdm09 virus isolated from a giant panda compared with other influenza viruses

Gene	Viruses showing highest similarity	Identity, %*
PB2	A/Wisconsin/629-D01313/2009	100
PB1	A/Sichuan/1/2009 (H1N1)	100
PA	A/Sichuan/1/2009 (H1N1)	100
HA	A/Sichuan/1/2009 (H1N1)	100
NP	A/Paris/2580/2009	100
NA	A/Sichuan/1/2009 (H1N1)	100
M	A/California/04/2009	100
NS	A/Sichuan/1/2009 (H1N1)	100

*Percent identity was calculated based on the complete open reading frames of each viral RNA segment.

Technical Appendix Table 2. Hemagglutination inhibition antibody titers of reference serum sample against influenza viruses of different subtypes*

Antigens†	H1N1/2009	H3	H5	H6	H7	H9
H1N1/2009	80	<10	<10	<10	<10	<10
H3	<10	160	<10	<10	<10	<10
H5	<2	<2	64	<2	<2	<2
H6	<2	<2	<2	64	<2	<2
H7	<2	<2	<2	<2	128	<2
H9	2	<2	<2	<2	<2	64

*Six reference serum samples were used in hemagglutination inhibiting serum antibody titers tests. The reference serum samples for H1N1/2009 and H3 seasonal influenza viruses were collected from ferrets and provided by the Chinese Center for Disease Control and Prevention. The reference serum samples for H5, H6, H7 and H9 subtype avian influenza viruses were collected from specific-pathogen-free chickens and provided by the Animal Influenza Laboratory of the Ministry of Agriculture, China.
†Six influenza virus subtypes were used in hemagglutination inhibiting serum antibody titers tests: H1N1/2009 (A/giant panda/01/Ya'An/2009), H3 (A/Victoria/361/2011 [H3N2]), H5 (A/duck/Anhui/1/2006 [H5N1]), H6 (A/Mallard/SanJiang/275/2007 [H6N1]), H7 (A/Baer's pochard/HuNan/414/2010 [H7N1]), and H9 (A/Chicken/Jinan/Li-2/2010 [H9N2]).

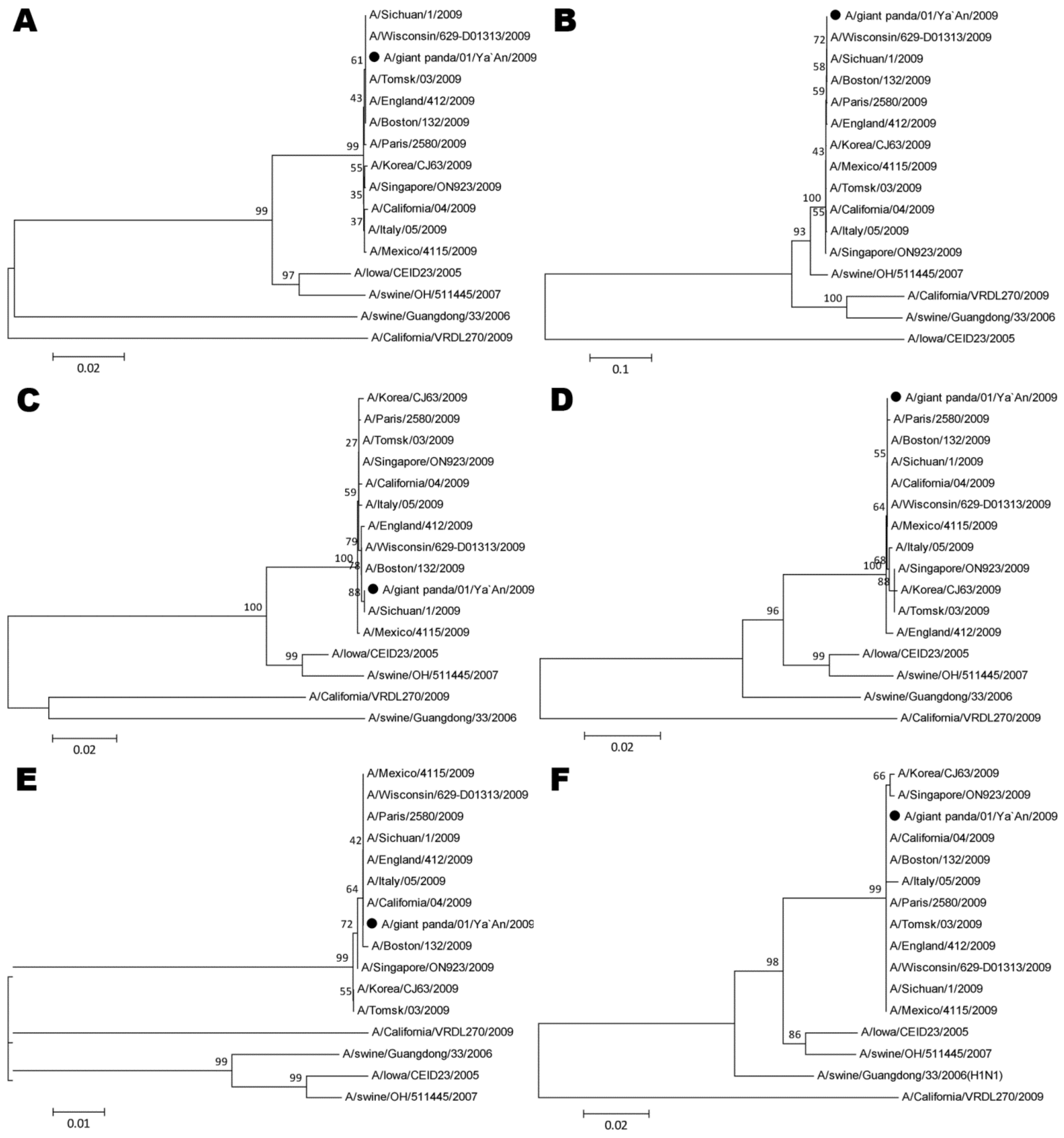

Technical Appendix Figure. Phylogenetic trees of influenza A(H1N1)pdm09 virus isolated from a giant panda in China compared with previously characterized pandemic influenza A(H1N1) viruses. A) PB2 gene nucleotide sequences. B) PB1 gene nucleotide sequences. C) PA gene nucleotide sequences. D) NP gene nucleotide sequences. E) M gene nucleotide sequences. F) NS gene nucleotide sequences. Neighbor-joining trees were created by using MegAlign software version 5.0 (www.megasoftware.net). Bootstrapping with 1,000 replicates was performed to determine the percentage reliability for each internal node. Horizontal branch lengths are proportional to genetic distances. Black dot indicates the isolate from this study. Scale bars indicate nucleotide substitutions per site.
